# Reutilization of Silty Sandstone Shield Spoil for Sustainable Synchronous Grouting: Mechanical Properties and Microstructure Characterization

**DOI:** 10.3390/ma19040778

**Published:** 2026-02-16

**Authors:** Changying Yu, Dong Yang, Shuishen Li, Yongfeng Wang, Changjie He, Yi Chen, Liangjie Zhan, Gongxun Wang

**Affiliations:** 1 China Construction Fifth Engineering Division Co., Ltd., Changsha 410004, China; 2 School of Civil Engineering, Hunan University of Science and Technology, Xiangtan 411201, China; 3 Hunan Province Intelligent Construction Assembly Passive House Engineering Technology Research Center, Xiangtan 411201, China

**Keywords:** shield muck, argillaceous siltstone, sodium trimethylsilanolate, hydration, pore structure

## Abstract

Conventional synchronous grouting materials often exhibit low early strength, delayed setting, and insufficient utilization of excavated soil, hindering the green and efficient advancement of metro shield tunneling technology. To overcome these challenges, this study developed a high-performance grouting material by utilizing shield muck—primarily composed of quartz (71.47%) and calcite (15.3%)—as the main raw material, with sodium trimethylsilanolate (TMS-Na) introduced as a performance enhancer. Through orthogonal experiments and range analysis, the influences of cement content, slag content, and TMS-Na dosage on the workability and mechanical properties of synchronous grouting materials were systematically evaluated. Microstructural evolution was characterized using scanning electron microscopy (SEM), X-ray diffraction (XRD), Fourier-transform infrared spectroscopy (FTIR), thermogravimetric (TG) analysis, and mercury intrusion porosimetry (MIP) to elucidate the mechanism by which TMS-Na modifies the grout microstructure. The results demonstrate that incorporating 8% slag and 0.2% TMS-Na increases the utilization rate of shield muck to 60.8%. Compared with conventional grouts, the novel material exhibits approximately 97.4% and 93.3% enhancements in 3-day and 28-day compressive strength, respectively, alongside an impermeability grade reaching P10. The addition of slag improves the apparent density and early strength of the grout, although its contribution diminishes at later ages. TMS-Na effectively activates the hydration reactivity of slag, accelerates early hydration, reduces the setting time, and participates in a secondary hydration reaction with argillaceous siltstone present in the excavated soil, promoting the formation of additional calcium silicate hydrate (C-S-H). This process densifies the hardened grout matrix, refines the pore structure, and significantly enhances both mechanical performance and impermeability. Field application in a trial tunnel section confirms that the proposed grouting material achieves complete cavity filling, eliminates water leakage, controls ground deformation effectively, and offers favorable economic viability, demonstrating strong potential for large-scale engineering application.

## 1. Introduction

The development and utilization of urban underground space represent a significant indicator of modern urban advancement. Shield tunneling, valued for its safety, efficiency, and minimal environmental impact, has become the predominant method for constructing linear underground infrastructure such as metro tunnels. However, this technique generates substantial amounts of excavated soil, commonly referred to as shield muck, with annual production in China alone exceeding 119 million tons [1]. Currently, most of this muck is disposed of through stockpiling or landfilling, leading to land occupation, dust pollution, ecological disruption, and considerable transportation and disposal costs [2,3,4,5,6,7,8].

Synchronous grouting is a critical procedure in shield tunneling, aimed at filling the annular tail void behind the shield to control ground deformation and settlement, thereby ensuring the stability of the segmental lining and the long-term safety of the tunnel [9,10,11,12,13]. Conventional synchronous grouting materials typically consist of cement, fly ash, bentonite, and sand [14,15,16]. Their production not only consumes significant natural resources and energy but also increases project costs and carbon emissions. Consequently, the development of high-performance grouting materials by utilizing solid wastes such as shield muck as substitutes for virgin materials is of considerable importance. This approach supports the transition of tunnel engineering towards green, low-carbon, and sustainable development by promoting waste recycling and resource recovery [17,18,19].

For example, Wang et al. [20] developed a novel in situ thick-layer grouting material using Chengdu metro shield muck, fly ash, cement, silica fume, and water glass. They observed that fly ash, water glass, and silica fume improved slurry stability, compressive strength, and impermeability. Under water dissolution and erosion conditions, its 28-day compressive strength was 0.58 MPa higher than that of pure cement paste, demonstrating favorable erosion resistance. However, the high silica fume content resulted in increased costs. Similarly, Cui et al. [21] formulated a specialized shield muck treatment agent composed of epoxy resin, a curing agent, and nano-silica. This agent bonds with soil particles and forms a polymeric material with polycrystalline characteristics at the interface, thereby enhancing the workability and mechanical properties of the grout. Nevertheless, its high cost limits large-scale application.

To reduce costs and improve the utilization rate of shield muck, Ni et al. [22] employed slag and shield slurry as precursors, activated by sodium sulfate and lime, to prepare a novel synchronous grouting material, which was successfully applied in the Nanjing Metro Line 6 project. Although it achieved a high 3-day compressive strength of 6.08 MPa, its prolonged setting time of 11.6 h significantly hindered construction efficiency, underscoring the need to balance material cost with workability performance.

Meanwhile, the adoption of optimization methods provides new avenues for enhancing muck utilization efficiency. Zhang et al. [23] used shield muck as a substitute for bentonite, blending it with river sand and cement to prepare grouting material. By applying response surface methodology and nonlinear regression analysis, they determined the optimal mix proportion that was subsequently implemented in the Zhengzhou Metro Line 3 project. This mix achieved a shield muck utilization rate of approximately 31% and a 28-day compressive strength of 2.54 MPa, though the underlying micro-mechanisms were not thoroughly examined. In another study, Zhang et al. [24] found that shield muck (silty sand and silty clay) could fulfill the roles of both natural sand and bentonite in synchronous grouting. Through regression analysis and mix optimization, the developed material attained a 28-day compressive strength of 3.86 MPa and a muck utilization rate of 50%. Zhao et al. [25] developed a geopolymer grouting material using shield muck, slag, fly ash, and sodium hydroxide, achieving a 28-day compressive strength of 4.12 MPa. However, this required a high sodium hydroxide content of 7%, and the muck utilization rate remained limited to only 30%, failing to fundamentally address the issues of high cost and low muck utilization.

In summary, the utilization rate of shield muck in synchronous grouting materials remains relatively low. Current research has predominantly focused on the physical and chemical properties of the muck and the macroscopic performance of grouting materials, such as workability and mechanical strength. There is a notable lack of systematic and in-depth investigation into the microstructural formation mechanisms and the action mechanisms of key chemical activators.

Argillaceous siltstone is a common constituent of shield muck encountered when tunneling through specific strata. Its use as a primary raw material for synchronous grouting presents several technical challenges: (1) low utilization rate; (2) adverse effects of contained clay minerals on the workability (e.g., fluidity, consistency) and setting characteristics of the slurry [26]; (3) considerable variations in mineral composition and physical properties (e.g., moisture content, particle gradation), leading to inconsistent performance of the grouting material [27]; and (4) insufficient understanding of the action mechanisms of chemical activators and their impact on material microstructure [28].

To address these challenges, this study aims to develop a high-performance synchronous grouting material using muck from the Changsha Metro Line 7 shield tunneling project as the main raw material, supplemented with ground granulated blast-furnace slag (GGBS) and TMS-Na as composite additives [29,30]. The effects of these composite additives on the workability, mechanical strength, hydration process, and microstructure of the shield muck-based grouting material are systematically investigated. The evolution characteristics of the pore structure and its relationship with macroscopic properties are elucidated. The material was successfully demonstrated in the Changsha Metro Line 7 shield project, achieving efficient utilization of shield muck. This work provides a theoretical foundation for advancing the application of a new generation of green, high-performance grouting materials in tunnel engineering.

## 2. Test Materials and Design

### 2.1. Material

Portland cement (P.O 42.5) was supplied by Hunan Shaofeng Nanfang Cement Co., Ltd. (Xiangtan, China). Its key properties include a specific surface area of 360 m^2^/kg, standard consistency water demand of 26.2%, initial setting time ≥ 150 min, final setting time ≤ 240 min, 28-day compressive strength ≥ 42.5 MPa, and 28-day flexural strength ≥ 6.5 MPa. Grade I Fly ash was obtained from Tianjin Zhucheng New Material Co., Ltd. (Tianjin, China), featuring specific surface area ≥ 1200 m^2^/kg and a 28-day activity index > 90%. Bentonite was produced by Henan Xinyang Zhongshan Thermal Insulation Building Materials Co., Ltd. (Xinxiang, Henan, China), the appearance of yellow and white, 1250 mesh. Grade S95 ground granulated blast-furnace slag (GGBS) was provided by Hunan Xincheng Mining Co., Ltd. (Changsha, China) with a specific surface area of 400 m^2^/kg, density ≥ 2.8 g/cm^3^, and loss on ignition ≤ 1%. Fine sand was produced from Hunan Wuxing sand field (Xiangtan, China) with fineness modulus ≥ 1.5, silt content < 5%, and particle size < 5 mm. TMS-Na, supplied by Shandong Yousuo Chemical Technology Co., Ltd. (Jinan, China) is a white powder with the main chemical composition being analytically pure (CH_3_)_3_SiONa. Table 1 shows the chemical composition of raw materials, as determined by X-ray fluorescence spectrometer.

### 2.2. Physical Properties of Shield Muck

**Figure 1:**
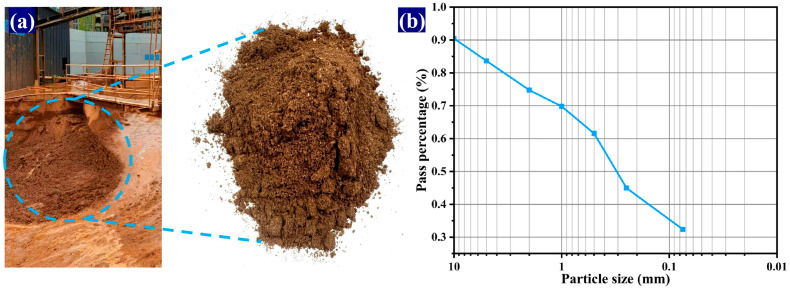
Shield muck and particle distribution size curve. (**a**) Shield muck; (**b**) Particle size distribution curve.

Filter-pressed shield muck collected from the construction site (Figure 1a) was oven-dried at 105 °C until a constant weight was attained. The dried material was subsequently crushed and sieved to obtain particles smaller than 2 mm, which were then stored for subsequent use. Figure 1b shows the particle size distribution curve of the muck. The physical indices of the shield muck (Natural water content: 17.07%, liquidity index: 0.12, plasticity index: 18.27) provide crucial guidance for the mix design. The medium-to-high plasticity index indicates the necessity of adding dispersants like TMS-Na to counteract the high-water demand caused by clay particles. The low liquidity index suggests that the muck itself is in a stable, plastic state, which is beneficial for mix proportion control. The well-defined natural water content ensures the accuracy of water-to-binder ratio calculations. These characteristics collectively dictate that a targeted composite activation and dispersion technical solution must be employed when utilizing this material as the primary aggregate.

**Figure 2:**
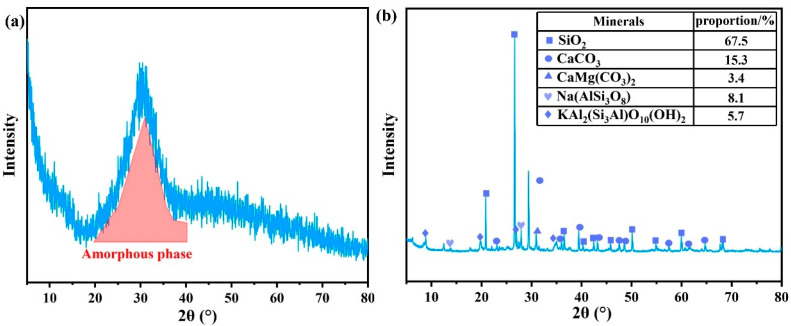
XRD mineral analysis of slag and shield muck. (**a**) GGBS; (**b**) Shield muck.

Figure 2 shows XRD mineral analysis of GGBS and the shield muck. The main active component of GGBS is the vitreous oxide, which belongs to the amorphous phase. It has a typical amorphous ‘steamed bread peak’ in the range of 20–40°, as shown in Figure 2a. Figure 2b identifies quartz, calcite, dolomite, muscovite, and albite as the dominant mineral phases in the shield muck.

### 2.3. Experimental Design

An orthogonal experimental design with four factors and three levels was employed to formulate the grouting materials. The factors included cement (*A*), slag (*B*), TMS-Na (*C*), and water (*D*). The levels for each factor were as follows: cement content at 7%, 8%, and 9%; slag content at 7%, 8%, and 9%; TMS-Na content at 0.1%, 0.15%, and 0.2%; and water content at 23%, 24%, and 25%. Accordingly, nine mix proportions were designed, as summarized in Table 2.

### 2.4. Specimen Preparation

The raw materials were batched according to the designed mix proportions. The slurry was prepared by initially mixing the shield muck and water for 60 s. cement and slag were then added and blended at a low speed for 90 s, after which the mixture was subjected to high-speed mixing for 30 s. Finally, TMS-Na was incorporated and mixed thoroughly for 120 s to produce the fresh slurry. Its workability, encompassing fluidity and setting time, was evaluated promptly. Subsequently, The slurry was then cast into 70.7 mm cubes and cured under standard conditions. Compressive strength was determined after 3 and 28 days of curing.

### 2.5. Test Methods

Fluidity was measured using an NLD-3 cement mortar fluidity tester (manufactured by Beijing Nerd Intelligent Technology Co., Ltd., Beijing, China) in accordance with Chinese standard JGJ/T 70-2009 [31]. Fresh slurry was placed into a truncated conical mold positioned centrally on the flow table, compacted, and leveled. After vertically lifting the mold, the table was jolted 25 times within 30 s. The fluidity was recorded as the average of the maximum spread diameters measured in two perpendicular directions.

Density was determined in accordance with the Chinese standard JGJ/T 70-2009. Freshly mixed slurry was placed into a standard container of known mass (*m*_1_) and volume (*V*), compacted as specified, and struck off level. The total mass (*m*_2_) was then measured. The slurry density (*ρ*) was calculated using Equation (1):

*ρ* = (*m*_2_ − *m*_1_)/*V* (1)

where *m*_1_ and *m*_2_ are the mass of the empty container and the mass of the container plus the slurry, respectively. The test was conducted in duplicate, and the arithmetic mean of the two results was taken as the final value.

Setting time was measured with a ZKA-100 mortar penetrometer (manufactured by Shanghai Meiyu Instrument and Equipment Co., Ltd., Shanghai, China) per Chinese standard JGJ/T 70-2009. Fresh slurry was cast into a metal mold (Ø140 × 175 mm), and cured at 20 ± 2 °C. Testing began after surface bleeding ceased. A 30 mm^2^ needle was penetrated 25 mm into the sample within 10 s, and the resistance was recorded. Initial testing intervals were 30 min, reduced to 15 min after the resistance reached 0.3 MPa, and continued until the resistance exceeded 0.7 MPa. The setting time was determined by interpolating the resistance-time curve to 0.5 MPa.

Compressive strength was determined using a WAW-100B testing machine (manufactured by Beijing Aerospace Road Construction Test Instrument Co., Ltd., Beijing, China) in accordance with Chinese standard JGJ/T 70-2009. Fresh slurry was cast into 70.7 mm^3^ cube molds and compacted on a vibration table. Specimens were demolded after 24 h and cured under standard conditions (20 ± 2 °C, relative humidity > 90%) until the target ages (e.g., 3 and 28 days). A constant loading rate of 0.25–1.5 kN/s was applied until failure. The final reported strength for each mixture represents the arithmetic mean of three specimens.

Impermeability was evaluated with an SS-15 mortar permeameter (manufactured by Hebei Cangzhou Tiantuo Instrument and Equipment Co., Ltd., Cangzhou, China) according to Chinese standard JGJ/T 70-2009. Slurry was cast into standard frustum-shaped molds (top diameter: 70 mm, bottom diameter: 80 mm, height: 30 mm). After standard curing to the specified age, a set of six specimens was mounted and sealed in the permeameter. The test started at a water pressure of 0.2 MPa, held for 2 h, and then increased by 0.1 MPa hourly until water penetration was observed on three specimens. The corresponding pressure (*H*) was recorded, And the impermeability pressure (*P*) was calculated using Equation (2), which determines the impermeability grade (e.g., P8, P10).

*P* = *H* − 0.1 (2)

Basic properties of shield muck, including natural water content, liquidity index, and plasticity index, were determined following Chinese standard GB/T 50123-2019 [32]. The natural water content was measured by the oven-drying method. A representative sample was weighed, dried at 105 °C to constant mass, and re-weighed, with the water content calculated from the mass loss ratio. The plasticity index and liquidity index were measured using an LP-100D liquid-plastic limit combined tester (manufactured by Shanghai Leiyun Test Instrument Manufacturing Co., Ltd., Shanghai, China). The plasticity index and liquidity index were then calculated by the standard formula in GB/T 50123-2019, based on the natural water content, liquid limit and plastic limit.

For microscopic examination, fractured specimens were trimmed into 1 cm cubes, vacuum-dried at 50 °C for 24 h, and sputter-coated with a thin gold layer prior to observation using a Zeiss Sigma 300VP (Carl Zeiss AG, Oberkochen, Germany) scanning electron microscope (SEM). The SEM was operated at accelerating voltages of 1–30 kV, probe currents ranging from 3 pA to 20 nA, a working distance of 5 mm, and an image acquisition size of 32 k × 24 k pixels. Pore structure characteristics were analyzed with a Micromeritics AutoPore IV 9500 (Micromeritics Instrument Corporation, Norcross, GA, USA) mercury intrusion porosimeter at 25 °C. The applied intrusion pressure range of 0.51–33,000 psi corresponded to a measurable pore-diameter range of 5.48 nm to 353.23 μm.

For mineralogical and phase analysis, the fractured specimens were ground into powder and passed through a 200-mesh sieve. The powder was vacuum-dried at 50 °C for 24 h. X-ray diffraction (XRD) patterns were acquired on a Rigaku Ultima IV diffractometer (Rigaku Corporation, Tokyo, Japan) with Cu-Kα radiation, scanning from 5° to 80° (2θ) at a rate of 10°/min. For chemical bond characterization, the powder samples were uniformly mixed with anhydrous KBr and pressed into transparent pellets. Fourier-transform infrared (FTIR) spectra were recorded on a PerkinElmer Spectrum Two spectrometer (PerkinElmer Enterprise Management Co., Ltd., Shanghai, China) in the range of 400–4000 cm^−1^ at a resolution of 2 cm^−1^. Thermogravimetric (TG) analysis was performed using a NETZSCH STA 449F3 thermal analyzer (Netzsch Instrument Manufacturing Co., Ltd., Selb, Germany) under a nitrogen flow of 250 mL/min, heating from 30 °C to 1050 °C at 20 °C/min.

## 3. Results and Discussion

### 3.1. Slurry Fluidity

**Figure 3:**
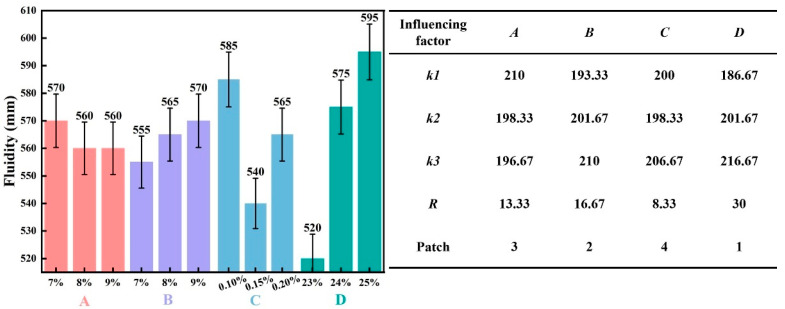
The influence of various influencing factors on slurry fluidity and range analysis.

The range analysis method was employed to calculate the sum of level indexes (K), the average level index (*k*), and the range (*R*). The effects of each influencing factor on the fluidity of the slurry and the corresponding range analysis are presented in Figure 3.

As shown in Figure 3, elevating the content of Factor *D* from 23% to 25% correspondingly raised the *k*-value from 186.67 to 216.67 mm, an increase of 30 mm, which confirms that slurry fluidity improves with higher water content. Increasing the content of Factor *B* from 7% to 9% resulted in an increase in the *k*-value from 193.33 to 210 mm (approximately +16.67 mm), indicating a beneficial effect of slag on fluidity. Similarly, a higher dosage of Factor *C* from 0.1% to 0.2% led to a modest rise in the *k*-value from 200 to 206.67 mm (+6.67 mm), reflecting its limited influence. In contrast, increasing the content of Factor *A* from 7% to 9% caused the *k*-value to decline from 210 to 196.67 mm (−13.33 mm), demonstrating that higher cement content reduces fluidity.

Thus, Factors *B* and *D* are positively correlated with slurry fluidity, whereas Factor *A* shows a negative correlation. Range analysis reveals that Factor *D* has the largest *R*-value (30), highlighting the dominant effect of water content on fluidity. The other factors exhibit relatively minor influences, with *R*-values ranging only between 8 and 17. Overall, the factors can be ranked in order of decreasing influence on slurry fluidity as: water > slag > cement > TMS-Na.

### 3.2. Slurry Density

**Figure 4:**
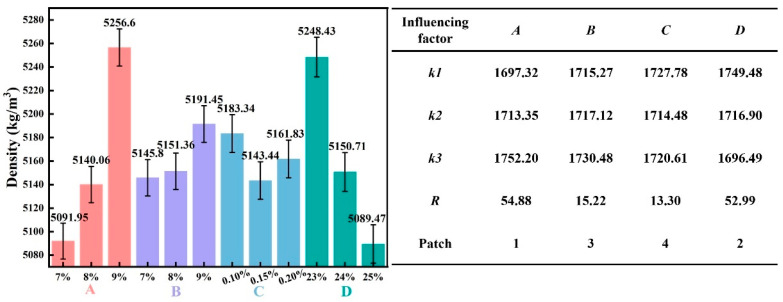
The influence of various influencing factors on slurry density and range analysis.

Figure 4 presents the effects of each influencing factor on the slurry density, along with the corresponding range analysis.

As shown in Figure 4, elevating the content of Factor *A* from 7% to 9% increased the *k*-value from 1697.32 to 1752.2 kg/m^3^ (+54.88 kg/m^3^), confirming that slurry density rises with higher cement content. Increasing the content of Factor *B* from 7% to 9% led to an increase in the *k*-value from 1715.27 to 1730.48 kg/m^3^ (+15.21 kg/m^3^), reflecting the positive role of slag in enhancing density. In contrast, a higher dosage of Factor *C* from 0.1% to 0.2% caused a slight decline in the *k*-value from 1727.78 to 1720.61 kg/m^3^ (−7.17 kg/m^3^), demonstrating its negligible effect on density. Similarly, increasing the content of Factor *D* from 23% to 25% reduced the *k*-value from 1749.48 to 1696.49 kg/m^3^ (−52.99 kg/m^3^), indicating that higher water content lowers slurry density.

Therefore, Factors *A* and *B* exhibit a positive correlation with slurry density, while Factor *D* shows a negative correlation. Range analysis indicates that Factor *A* has the largest *R*-value (54.88), followed by Factor *D* (*R* = 52.99), highlighting the dominant effects of cement and water content on density. The remaining factors show relatively weak influences, with *R*-values ranging between approximately 13 and 16. Overall, the factors can be ranked in order of decreasing influence on slurry density as: cement > water > slag > TMS-Na.

### 3.3. Setting Time of Slurry

**Figure 5:**
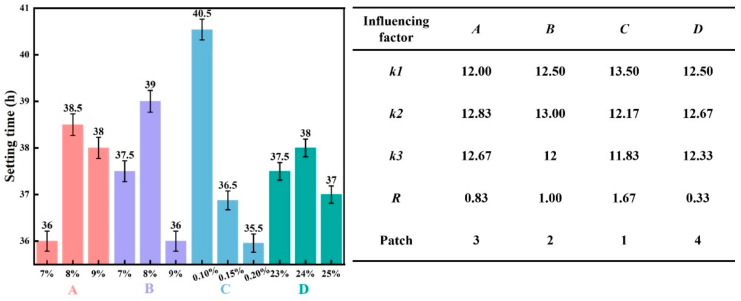
The influence of various influencing factors on the setting time of slurry and range analysis.

Figure 5 presents the effect of each factor on the slurry setting time and the corresponding range analysis.

As shown in Figure 5, increasing the content of Factor *C* from 0.1% to 0.2% resulted in a decrease in the *k*-value from 13.5 to 11.83 h (−1.67 h), indicating that a higher TMS-Na dosage shortens the setting time. This acceleration is attributed to the enhanced polymerization rate of the alkali-activated slag system by TMS-Na, which promotes the formation of reaction products and thus accelerates setting. Similarly, increasing the content of Factor *D* from 23% to 25% led to a minor decrease in the *k*-value from 12.5 to 12.33 h (−0.17 h), reflecting its limited influence. Increasing Factor *B* content from 7% to 9% reduced the *k*-value from 12.5 to 12 h (−0.5 h), suggesting that higher slag content also promotes faster setting. In contrast, a higher content of Factor *A* from 7% to 9% increased the *k*-value from 12 to 12.67 h (+0.67 h), demonstrating that cement retards the setting process.

Therefore, Factor *C* exhibits a negative correlation with the slurry’s setting time. The range analysis reveals that Factor *C* has the largest *R*-value of 1.67, followed by Factor *B*, confirming that both TMS-Na and slag content exert a significant influence on the setting time. In comparison, Factor *D* has the smallest *R*-value of 0.33, denoting its minimal effect. Overall, the factors are ranked in order of decreasing influence on setting time as: TMS-Na > slag > cement > water.

### 3.4. Slurry Compressive Strength

**Figure 6:**
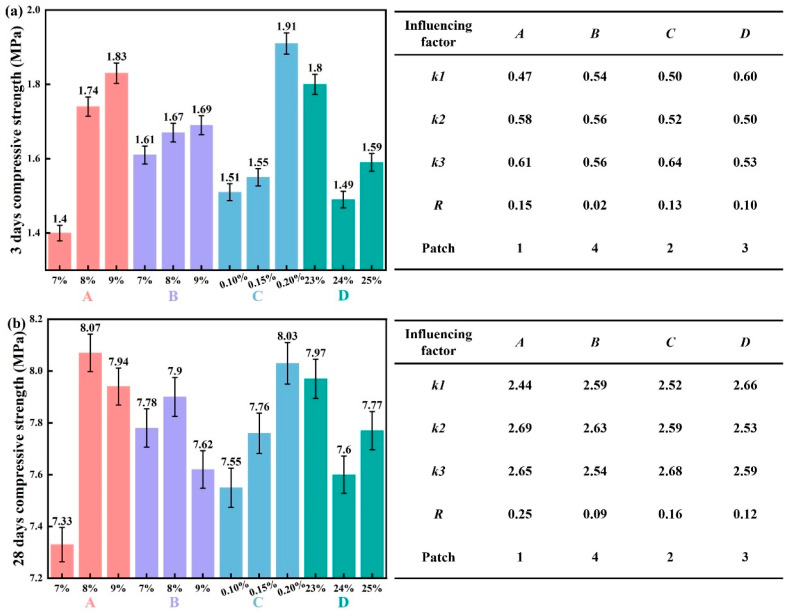
The influence of various influencing factors on the 3-day and 28-day compressive strength of the slurry and the range analysis. (**a**) 3 day; (**b**) 28 day.

Figure 6 shows the effects of various factors on the 3-day and 28-day compressive strength of the synchronous grouting material, along with the corresponding range analysis.

As shown in Figure 6a, increasing the content of Factor *A* from 7% to 9% raised the *k*-value from 0.47 to 0.61 MPa (+0.14 MPa), demonstrating an enhancement in the 3-day compressive strength. In contrast, as the content of Factor *B* increased from 7% to 9%, the *k*-value increased only slightly from 0.54 to 0.56 MPa, a marginal gain of 0.02 MPa, suggesting that increasing slag content has a limited effect on enhancing the 3-day compressive strength. A higher dosage of Factor *C* increased from 0.1% to 0.2% increased the *k*-value from 0.5 to 0.64 MPa (+0.14 MPa). Conversely, increasing Factor *D* from 23% to 25% reduced the *k*-value from 0.6 to 0.53 MPa (−0.07 MPa). Thus, Factors *A*, *B*, and *C* show a positive correlation with the 3-day compressive strength. The range analysis reveals that Factor *A* has the largest *R*-value of 0.15, followed by Factor *C* with an *R*-value of 0.13, highlighting their significant influence, whereas Factor *B* has a minimal *R*-value (0.02). Therefore, the factors are ranked by influence on 3-day strength as: cement > TMS-Na > water > slag.

From Figure 6b, increasing Factor *A* content from 7% to 9% elevated the *k*-value from 2.44 to 2.65 MPa, corresponding to a 0.21 MPa increase in 28-day compressive strength. Similarly, a higher dosage of Factor *C* from 0.1% to 0.2% increased the *k*-value from 2.52 to 2.68 MPa (+0.16 MPa). However, increasing Factor *B* from 7% to 9% slightly reduced the *k*-value from 2.59 to 2.54 MPa (−0.05 MPa), and increasing Factor *D* from 23% to 25% decreased it from 2.66 to 2.59 MPa (−0.07 MPa). Therefore, Factors *A* and *C* exhibit a positive correlation with the 28-day compressive strength. The *R*-value is largest for Factor *A* (0.25), followed by Factor *C* (0.16), confirming their dominant roles, while Factor *B* has a relatively small *R*-value (0.09). According to the ranking results, the degree of influence of each factor on the 28-day compressive strength, in descending order, is: cement > TMS-Na > water > slag.

Furthermore, increasing the dosage of TMS-Na contributes to improving both the 3-day and 28-day compressive strengths to a certain extent. This is attributed to the reactive silica in TMS-Na, which dissolves and participates in the formation of calcium silicate hydrate (C-S-H) and calcium aluminosilicate hydrate (C-A-S-H) gels, thereby promoting polymerization and strength development.

Based on this analysis, with compressive strength as the key indicator, the optimal factor levels were selected as A2 (8% cement), B2 (8% slag), C3 (0.2% TMS-Na), and D1 (23% water).

**Figure 7:**
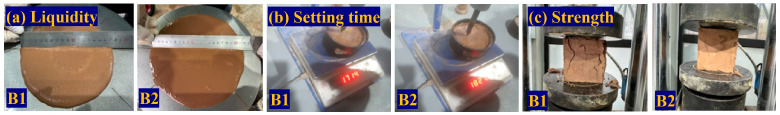
Comparison of various performance parameters.

**Figure 8:**
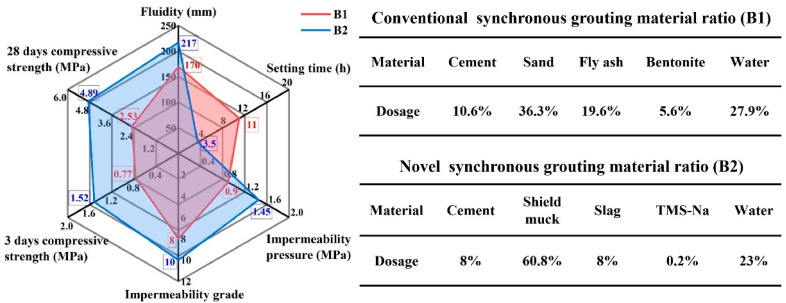
Comparison of performance between novel synchronous grouting materials and conventional synchronous grouting materials.

The performance of the novel synchronous grouting material with this optimal mix proportion was compared with that of the conventional synchronous grouting material. The detailed test parameters are presented in Figure 7, and the comparison results are shown in Figure 8.

As shown in Figure 8, compared to the conventional synchronous grouting material, the novel synchronous grouting material exhibits a 47 mm increase in fluidity, and its setting time is shortened from 11 h to 3.5 h. Its 3-day and 28-day compressive strengths are approximately 97.4% and 93.3% higher, respectively, than those of the conventional material. The impermeability pressure is increased by 0.55 MPa, raising the impermeability grade from P8 to P10. These results demonstrate that the combined incorporation of slag and TMS-Na can improve the fluidity, shorten the setting time, and enhance the strength and impermeability of the synchronous grouting material. This not only improves construction efficiency but also significantly increases the effective utilization rate of shield muck to 60.8%.

### 3.5. SEM Microanalysis

**Figure 9:**
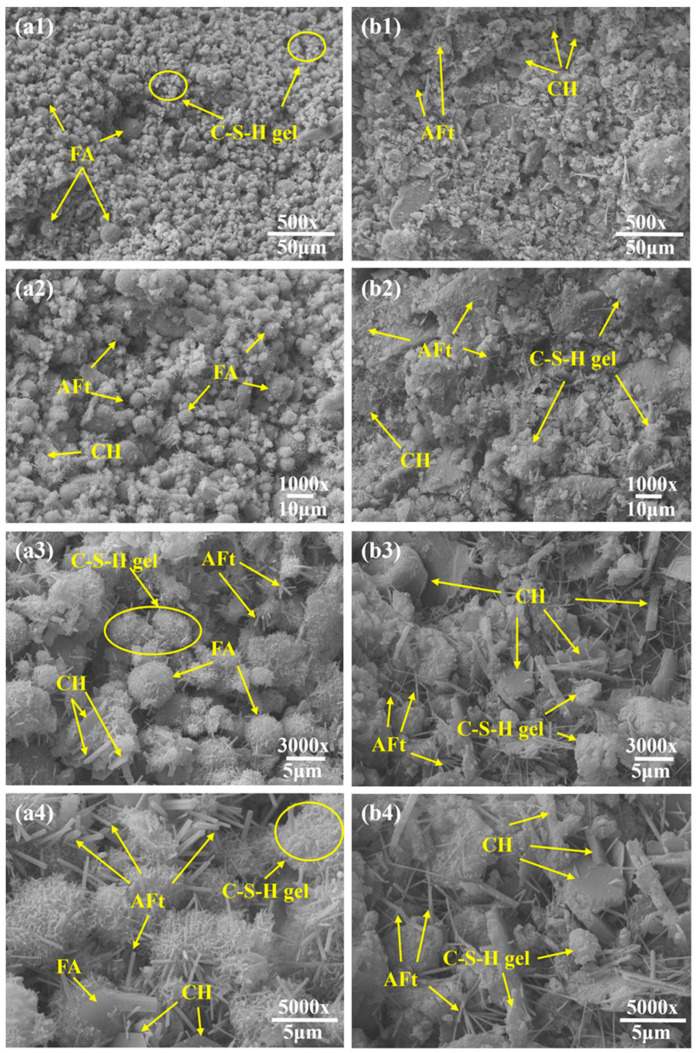
3-day SEM image. (**a1**–**a4**) Conventional synchronous grouting materials; (**b1**–**b4**) Novel synchronous grouting material.

**Figure 10:**
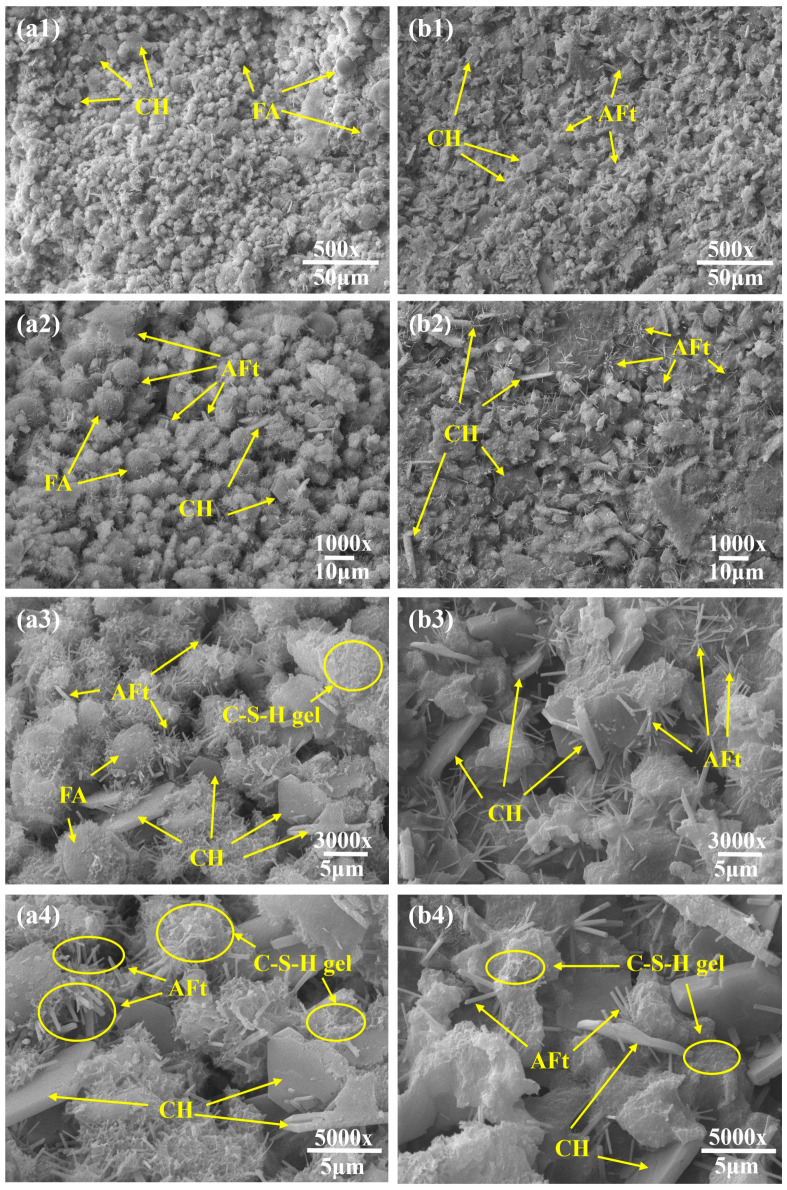
28-day SEM image. (**a1**–**a4**) Conventional synchronous grouting materials; (**b1**–**b4**) Novel synchronous grouting material.

The 3-day and 28-day specimens of the conventional synchronous grouting material and the novel synchronous grouting material were subjected to SEM microstructural analysis, with the results shown in Figure 9 and Figure 10, respectively.

As shown in Figure 9, at 3 days of hydration, the conventional material exhibited a relatively loose structure with unevenly distributed hydration products. Numerous loosely packed fly ash (FA) particles were present, with only a small amount of flocculent calcium silicate hydrate (C-S-H) gel formed on their surfaces (Figure 9(a1,a2)), indicating initial but limited participation of FA in the hydration reaction. Locally aggregated, hexagonal-plate Ca(OH)_2_ crystals (CH) smaller than 5 μm were occasionally observed. Additionally, uniformly distributed, whisker-like ettringite (AFt) needles, generally shorter than 5 μm, were visible (Figure 9(a3,a4)). In contrast, the novel grouting material displayed a dense, crack-free structure (Figure 9(b1,b2)). Its surface was covered with abundant needle-rod ettringite crystals, mostly longer than 5 μm, along with well-developed hexagonal-plate CH crystals exceeding 5 μm in size. These crystals were coated with flocculent C-S-H gel (Figure 9(b3,b4)), reflecting a more advanced stage of hydration.

After 28 days (Figure 10), most FA particles in the conventional material had participated in hydration and were covered by C-S-H gel, though some unreacted particles remained, resulting in loose packing and large interparticle pores, and the overall structure remained relatively porous. Aggregated CH crystals (≈5 μm) with smooth surfaces were observed and the C-S-H gel network was interspersed with numerous needle-rod ettringite crystals about 2 μm in length. Conversely, the novel grouting material exhibited a much denser micro-network structure with finer pores. The surface was densely populated with needle-rod ettringite crystals (≈5 μm) and larger hexagonal-plate CH crystals (≈10 μm), their surfaces also coated with C-S-H gel, indicating a more advanced hydration degree. Wu et al. [33] observed a similar micro-network structure in their study on the effects of slurry SG (an additive formed from shield mud and water) and binder content on the performance of a novel grouting material. They reported that with extended curing, the amounts of hydration products (ettringite and C-S-H gel) gradually increased, exhibiting an intergrown pattern that further filled microscopic pores, densified the structure, and consolidated loose shield mud into a framework-like configuration.

The reaction mechanisms in alkali-activated cementitious systems are well-established. It can be reasonably inferred that TMS-Na participated in the reactions as an alkali activator through a mechanism similar to that of other activators. Specifically, TMS-Na hydrolyzes to produce sodium hydroxide, which generates a strongly alkaline environment that disrupts the glassy structure of the slag, releasing internal silicate (${SiO}_{4}^{4-}$) and aluminate (${AlO}_{4}^{5-}$) ions. These ions subsequently undergo pozzolanic reactions with Ca^2+^ in the system, forming calcium silicate hydrate (C-S-H) and calcium aluminosilicate hydrate (C-A-S-H) gels that fill pores and enhance mechanical properties. This inference is supported by the experimental results of Yang et al. [34], who provided a similar mechanistic explanation. In addition, the hydrolysis of TMS-Na generates reactive trimethylsilanol ((CH_3_)_3_SiOH), whose silanol (Si-OH) groups condense with -OH groups on dissolved silicate or aluminate ions, forming stable Si-O-Si or Si-O-Al covalent bonds. This process is analogous to the mechanism described by Bondar et al. [35] for the dissolution of active mineral ions from aluminosilicate structures in fly ash using sodium silicate. Essentially, it can be regarded as the use of organic silicon “chains” [(CH_3_)_3_Si-O-] to cross-link the originally inorganic hydration product particles, creating an organic-inorganic hybrid, denser, and more robust three-dimensional network structure, which significantly improves the material’s integrity and mechanical performance. Simultaneously, the slag contributes through a micro-aggregate effect, some incompletely reacted or partially polymerized organosilicon compounds, along with by-products generated during the reaction, can fill microscopic pores, making the structure even denser and notably enhancing the material’s impermeability [36].

In summary, the novel composite activator promotes intense hydration through alkaline activation and silanol-based cross-linking. The resulting hydration products cement shield muck particles into agglomerates, roughen their surfaces, and rearrange them into a stable 3D network reinforced by C-S-H gel [37], thereby significantly improving mechanical strength and impermeability.

### 3.6. XRD Test

**Figure 11:**
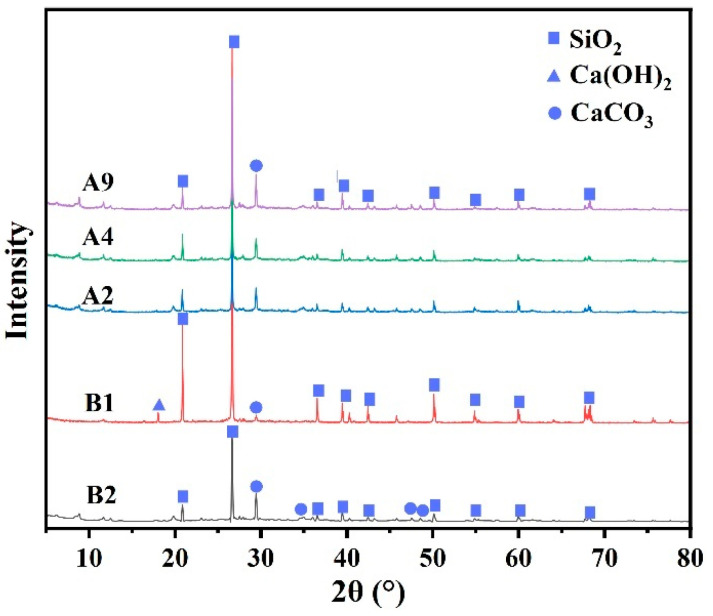
XRD patterns of conventional synchronous grouting materials and novel synchronous grouting materials.

Figure 11 presents the XRD patterns of the slurry mixtures after 28 days of hydration.

As shown in Figure 11, the main mineral phases identified in all mixtures are SiO_2_, Ca(OH)_2_, and CaCO_3_. Due to the high proportion of silty fine sand in the slurries, a strong diffraction signal corresponding to SiO_2_ is observed in every group. A comparison between the conventional (B1) and novel (B2) grouting materials shows that while both exhibit an intense SiO_2_ peak from the aggregate, its intensity is noticeably lower in B2. This attenuation suggests a more effective dilution and coating of the inert sand by hydration products in B2, which indirectly signifies a greater formation of C-S-H gel.

Notably, a weak Ca(OH)_2_ diffraction peak is present in B1, whereas no distinct peak is detected for B2. This absence can be ascribed to the strong activation of slag by TMS-Na in B2, which accelerates early hydration and consumes part of the Ca(OH)_2_ through secondary slag reactions. Moreover, a significant portion of the newly formed Ca(OH)_2_ is incorporated as nano-crystalline particles within the abundant C-S-H gel network. Owing to their poor crystallinity and extremely minute size, these Ca(OH)_2_ nano-crystals are difficult to detect by XRD [38]. Only in locally less reactive regions can a small amount of Ca(OH)_2_ aggregate and grow into well-crystallized hexagonal-plate crystals, as observed in the SEM images. This unique distribution—dominated by nano-crystals with occasional larger crystals—indicates higher reactivity and a more advanced hydration degree in the B2 system. The encapsulation of highly reactive nano-scale Ca(OH)_2_ within the C-S-H gel further promotes pozzolanic reactions [39], collectively leading to a denser microstructure.

Furthermore, the diffraction features of CaCO_3_ also support the above differences. In B1, only one distinct CaCO_3_ diffraction peak appears at 29.4°, whereas in B2, the peak at this position is stronger, and weak CaCO_3_ diffraction peaks emerge at several other angles. This indicates that carbonation is more extensive in B2, yielding a larger quantity and broader distribution of finely crystalline CaCO_3_. Additionally, the CaCO_3_ diffraction intensities in B2 are higher than those in groups A2, A4, and A9 at multiple angles, confirming its status as the optimal mix.

In summary, XRD phase analysis provides crystallographic evidence that the novel synchronous grouting material (B2) achieves more complete hydration, generating abundant C-S-H gel and Ca(OH)_2_, thereby establishing a robust microstructural foundation for its superior macroscopic performance.

### 3.7. TG Test

**Figure 12:**
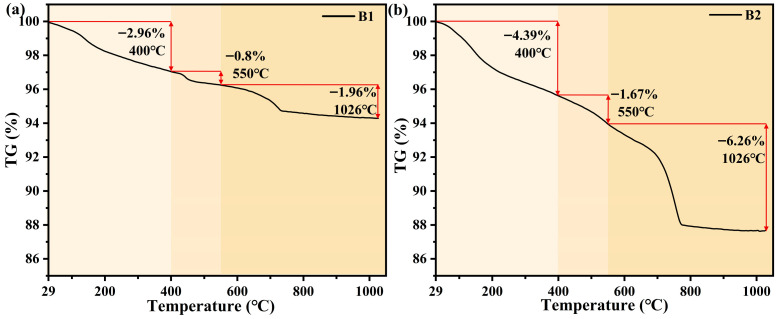
TG curves of conventional synchronous grouting materials and novel synchronous grouting materials. (**a**) Conventional synchronous grouting materials; (**b**) Novel synchronous grouting materials.

TG analysis was conducted after 28 days of hydration to quantitatively compare the hydration processes of the conventional and novel synchronous grouting materials. The results are presented in Figure 12.

The TG curves indicate that the thermal decomposition of both grouting materials occurs in three main stages: Stage 1 (29–400 °C), corresponding primarily to the dehydration of hydration products such as C-S-H gel and ettringite; Stage 2 (400–550 °C), attributed to the dehydroxylation of Ca(OH)_2_; and Stage 3 (550–1026 °C), associated with CaCO_3_ decarbonation.

For the conventional material, the mass losses rate are 2.96% (Stage 1) and 0.8% (Stage 2). This indicates a low content of Ca(OH)_2_ and a relatively limited degree of hydration. In the Stage 3, the mass loss rate is 1.96%, reflecting a low CaCO_3_ content and modest carbonation.

In contrast, the novel synchronous grouting material exhibits significantly greater mass losses across all stages: +1.43%, +0.87%, and +4.3%, respectively. Specifically, its mass loss reaches 4.39% in Stage 1, demonstrating more complete hydration and the formation of greater amounts of C-S-H gel and ettringite. The Stage 2 loss increases to 1.67%, confirming a significantly higher Ca(OH)_2_ content. The markedly higher loss in Stage 3 (total increase of 4.3%) reveals a more advanced degree of carbonation. This results from its higher Ca(OH)_2_ content and the larger specific surface area provided by the more developed gel system, which promotes fuller contact and reaction with atmospheric CO_2_ to form CaCO_3_.

In summary, TG analysis quantitatively confirms, from the perspective of thermal decomposition, that the novel synchronous grouting material achieves a higher degree of hydration. The amounts of C-S-H gel, Ca(OH)_2_, and CaCO_3_ in the novel synchronous grouting material are significantly greater than those in the conventional synchronous grouting material. These findings align well with the microstructural observations from SEM.

### 3.8. FTIR Test

**Figure 13:**
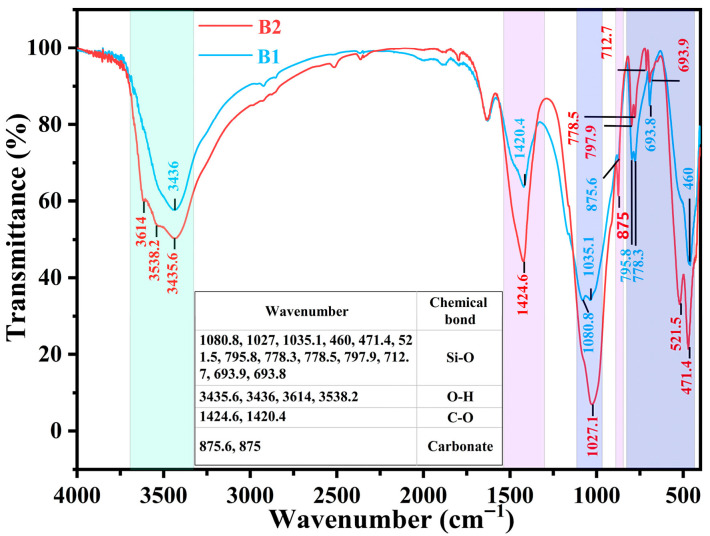
FTIR test of conventional synchronous grouting materials and novel synchronous grouting materials.

FTIR spectroscopy was employed to compare the chemical structures and reaction extent of the conventional (B1) and novel (B2) synchronous grouting materials after 28 days of hydration. The spectra are shown in Figure 13.

In the main Si-O stretching vibration region (1300–400 cm^−1^), B1 exhibits a doublet at 1080.8 cm^−1^ and 1035.1 cm^−1^, assigned to unreacted quartz (SiO_2_) and the asymmetric stretching vibration of Si-O bonds in C-S-H gel, respectively. In contrast, B2 exhibits a merged, broadened, and intensified single peak at 1027.1 cm^−1^, slightly shifted to a lower wavenumber. This indicates that more thorough pozzolanic and silicate polycondensation reactions occurred in the B2 system, driven by the reactive Si–Al sources from slag and TMS-Na. Consequently, the characteristic Si-O peak of unreacted SiO_2_ was obscured, reflecting the incorporation of inert sand into the hydration process and improved chemical homogeneity.

In the characteristic α-quartz region (795.8 cm^−1^, 778.3 cm^−1^, and 693.8 cm^−1^), B1 displays sharp absorption peaks, typical of the symmetric Si-O stretching vibration of crystalline α-quartz. In the spectrum of B2, these peaks are markedly weakened, slightly shifted, or even split: the peaks at 795.8 cm^−1^ and 778.3 cm^−1^ shift to 797.9 cm^−1^ and 778.5 cm^−1^, respectively, and the single peak at 693.8 cm^−1^ splits into a doublet at 712.7 cm^−1^ and 693.9 cm^−1^. These changes further confirms that the quartz derived from the shield muck in B2 underwent strong physical–chemical disturbance. Extensive encapsulation by C-S-H gel and the highly alkaline environment eroded its surface crystal structure, transforming it from an inert component into a phase that actively participates in the hydration reaction.

In the Si-O bending vibration region (1000–400 cm^−1^), the sharp single peak at 460 cm^−1^ in B1, representing crystalline quartz, is completely replaced in B2 by broadened double peaks at 471.4 cm^−1^ and 521.5 cm^−1^, with significantly enhanced absorption intensity. This further demonstrates that the shield muck aggregate in B2 is not an inert filler. Its quartz crystal structure was severely eroded and disrupted under the strong alkalinity provided by the slag and the likely catalytic environment of TMS-Na. Simultaneously, large amounts of amorphous C-S-H and C-A-S-H gels were formed in the system. The Si-O vibrations in these gel phases give rise to the broadened doublets [40].

In the hydroxyl stretching region (4000–3000 cm^−1^), the absorption peaks at 3435.6 cm^−1^ (B2) and 3436 cm^−1^ (B1) are mainly attributed to the O-H stretching vibration of physically adsorbed water. The higher absorption intensity of the broad peak at 3435.6 cm^−1^ in B2 indicates a greater content of adsorbed water within its structure, smaller pores, and stronger capillary adsorption and water retention capacity. Moreover, B2 exhibits two distinct low-intensity peaks at 3614 cm^−1^ and 3538.2 cm^−1^, which are characteristic O-H stretching vibrations of well-crystallized Ca(OH)_2_. This phenomenon suggests intense early cement hydration in B2, generating a substantial amount of Ca(OH)_2_ to support subsequent slag activation.

In the carbonate region, the out-of-plane bending vibration near 875 cm^−1^ is stronger in B2, indicating more extensive carbonation. This indirectly supports the higher content of Ca(OH)_2_ hydration products in B2, which have a larger specific surface area and are more prone to react with atmospheric CO_2_ to form CaCO_3_. The absorption peaks at 1420.4 cm^−1^ (B1) and 1424.6 cm^−1^ (B2) belong to the asymmetric C-O stretching vibration of ${CO}_{3-}^{2-}$. The slight blue shift may indicate that the formed CaCO_3_ has higher crystallinity or a different crystal form (e.g., subtle differences between calcite and aragonite). More importantly, it reflects the perturbation of the chemical environment around the carbonate, typically due to its encapsulation by denser C-S-H gel. The pronounced intensity increase directly corresponds to a greater quantity of ${CO}_{3-}^{2-}$ ions, confirming more extensive carbonation in B2 as a result of its higher Ca(OH)_2_ content and advanced hydration—findings consistent with TG analysis.

In summary, the synergistic effect of slag and TMS-Na in the novel synchronous grouting material significantly accelerates and deepens the hydration process. It not only yields more highly polymerized C-S-H gel but also effectively activates and utilizes the inert shield muck aggregate, achieving overall optimization and homogenization of the system’s chemical structure. Thus, FTIR analysis provides a fundamental chemical explanation for the enhanced macroscopic mechanical performance of the novel material.

### 3.9. MIP Test

**Figure 14:**
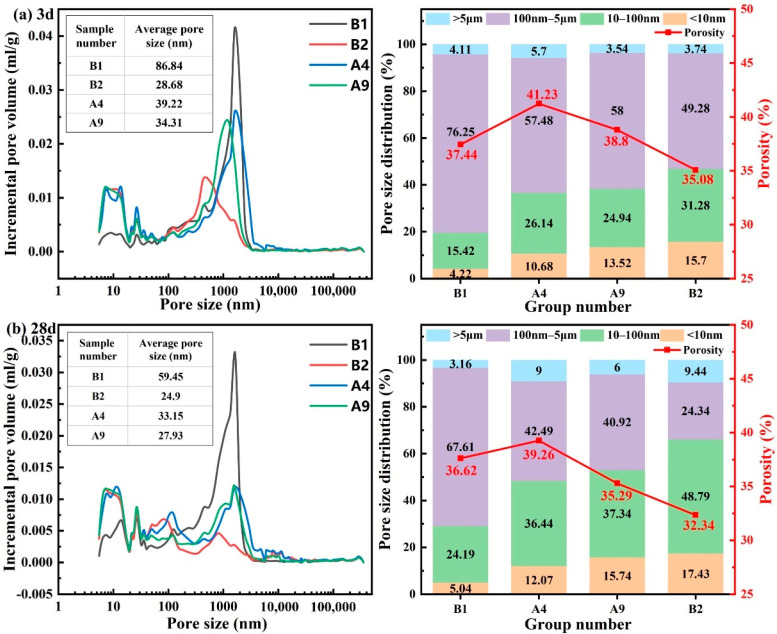
The pore size distribution curve and pore volume percentage accumulation diagram of different grouting material ratios. (**a**) 3-day; (**b**) 28-day.

Pore structure serves as a key indicator for evaluating the durability and mechanical performance of cement-based materials. Its distribution characteristics directly determine the material’s compactness and permeability, thereby affecting its mechanical strength and impermeability. To evaluate the effects of hydration age and mix design on the pore structure of grouting materials, MIP was performed on various slurry mixtures after 3 days and 28 days of hydration. The results are summarized in Figure 14.

As shown in Figure 14a (3 days), the pore structures of the four mix proportions exhibited a graded difference, with B2 performing the finest porosity. In terms of total porosity, B1 had the highest value (37.44%), while B2 had the lowest (35.08%). A4 and A9 fell between these two, indicating that the novel synchronous grouting materials generally achieved a denser early-age structure. Average pore size differed even more markedly: B1 had the largest size (86.84 nm), while B2 showed the smallest (28.68 nm)—a reduction of about 67%. The values for A4 and A9 fell between those of B1 and B2, underscoring the superior pore-refining capability of the novel materials. Regarding pore size distribution, B1 was dominated by mesopores (100 nm–5 μm; 76.25%), with only 4.22% micropores (<10 nm). In sharp contrast, B2 presented substantially higher proportions of intermediate pores (10–100 nm; 31.28%) and micropores (<10 nm; 15.70%), totaling ~46.98%. A4 and A9 also displayed higher intermediate- and micro-pore shares than B1 but lower than B2, indicating an intermediate level of structural refinement. Thus, after only 3 days, B2 had already developed a dense, fine-pore-dominated system.

With extended curing to 28 days (Figure 14b), all groups showed a trend of pore refinement, but the novel synchronous grouting material B2 exhibited the most pronounced optimization. Porosity decreased most for B1 (−2.74%) and least for B2 (−0.82%); A4 and A9 decreased by 1.97% and 3.51%, respectively. Nevertheless, the final porosity of B2 remained the lowest, highlighting its consistent densification advantage. The average pore size of B1 dropped considerably from 86.84 to 59.45 nm, whereas B2 decreased only slightly from 28.68 to 24.90 nm (A4 and A9 reached 33.15 and 27.93 nm). This trend suggests that B2, having already achieved a very fine pore system early, had limited scope for further reduction, while the other groups possessed greater “optimization potential.” Even so, B2’s final average pore size (24.9 nm) was markedly lower than those of the other groups. Regarding the evolution of pore size distribution, all groups showed a reduction in the proportion of mesopores and an increase in the proportions of intermediate pores and micropores. Among them, B2 exhibited the largest increase in intermediate-pore proportion (+17.51%), which was about 107% of the increase in B1 (+8.77%); A4 and A9 also exceeded B1. By 28 days, the pore structure of B2 was absolutely dominated by intermediate and micropores (combined proportion >66.22%), demonstrating markedly better refinement and uniformity.

Focusing on the optimal group B2, the specific evolution of its pore structure from 3 to 28 days clearly illustrates a continuous and efficient optimization process: the volume of mesopores decreased markedly, while the proportion of intermediate pores increased substantially (17.51%). This “pore transformation” directly confirms the continuous generation of hydration products (such as C-S-H gel), which progressively fill and segment larger pores. Although the absolute decrease in average pore size for B2 (3.78 nm) was smaller than that for B1, this results from the already ultrafine structure of B2 at early ages. This superior performance originates from its mix design: highly reactive slag promotes the formation of dense composite hydration products; TMS-Na acts as a hydrophobic agent and pore refiner, improving particle dispersion and the uniformity of hydration product precipitation. Its hydrolysis product (silanol) can reduce early water loss and fill pores [41]. Together, these mechanisms build and continuously refine a high-density, fine-pore system, providing the microstructural basis for the material’s outstanding mechanical performance.

## 4. Engineering Application

### 4.1. Shield Muck Dewatering Pretreatment and Pulping

**Figure 15:**
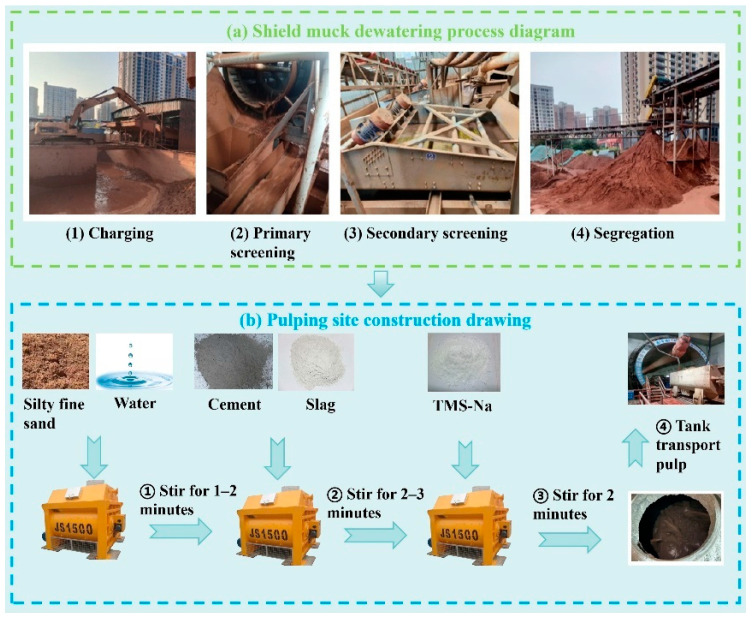
Shield muck dewatering pretreatment process and slurry site construction process. (**a**) Shield muck dewatering pretreatment process. (**b**) slurry site construction process.

An engineering-application trial was carried out on the Changsha Metro Line 7 project, utilizing shield muck from a designated tunnel section. The dehydration pretreatment process for the shield muck is shown in Figure 15a. A crucial stage involves using a double-layer shaftless rotary screen to separate the muck into coarse particles (>6 mm) and a sandy slurry fraction (<6 mm). The sandy slurry (<6 mm) then undergoes secondary sieving to finally obtain fine sand with a particle size below 2 mm. The on-site slurry preparation process is shown in Figure 15b.

### 4.2. Construction Effect Appraisal

**Figure 16:**
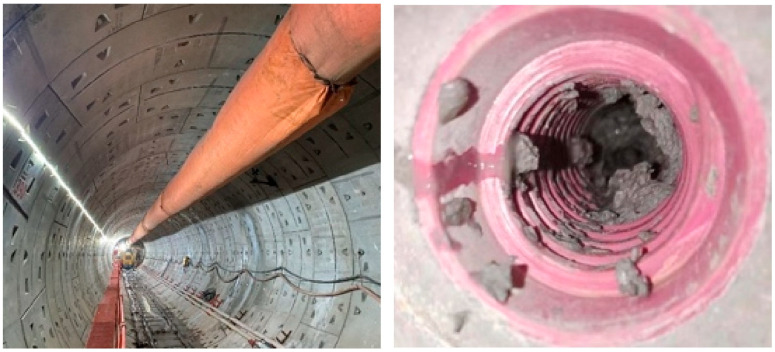
Test section and pipe opening inspection.

On the left line (rings No. 391–No. 393, constructed with conventional grout), the average grouting pressure was 0.3 MPa. In the corresponding test section on the right line (rings No. 391–No. 393, using the novel shield-muck-based grout), the pressure was 0.24 MPa. No construction issues, such as pipe blockage, were encountered, and segment quality remained satisfactory, demonstrating that the novel material is fully compatible with conventional grouting practices. Post-construction inspection of the test-section segments (Figure 16) revealed dense, complete grout filling without any leakage.

**Figure 17:**
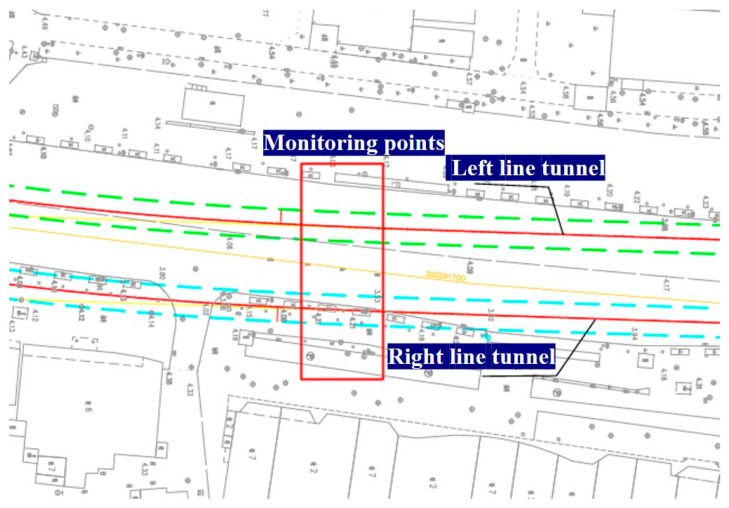
Layout of monitoring points.

**Figure 18:**
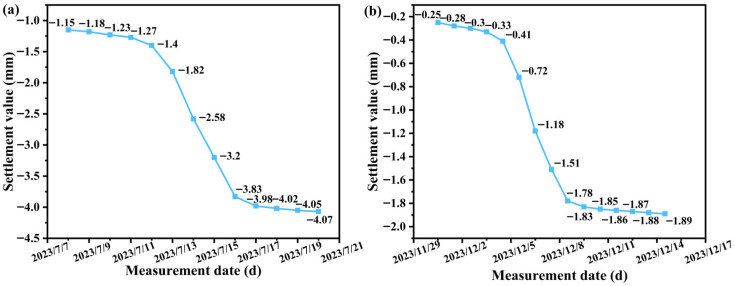
Settlement curve of excavated section. (**a**) Left line; (**b**) Right line.

Surface deformation was monitored along both tunnel alignments following shield passage to assess the effect of conventional versus novel grouting materials on ground settlement. The arrangement of monitoring points in the test section is shown in Figure 17, and the corresponding settlement curves for the left line (conventional-grout section) and the right line (novel-grout test section) are presented in Figure 18.

At ring No. 392, cumulative surface settlement before the segment left the shield tail was about 1.2 mm for the left line and only 0.4 mm for the right line. After tail passage, settlement increased to a maximum of 4.07 mm on the left line, whereas it reached only 1.89 mm on the right line.

These results demonstrate that the shield-muck-based novel grouting material reduces ground deformation significantly compared with the conventional material, with all recorded settlements lying well within the allowable limits specified in construction acceptance standards.

### 4.3. Economic Benefit Analysis

As summarized in Table 3, for the Changsha Metro Line 7 project, the unit cost of the conventional synchronous grouting material is 305.4 RMB/m^3^, whereas the cost of the novel synchronous grouting material is 89.96 RMB/m^3^. This represents a cost reduction of approximately 71% per cubic meter. If the remaining 692 rings of segments in the entire line were all constructed with the novel material, an estimated material cost saving of about 150,000 RMB could be achieved.

Furthermore, adopting conventional off-site disposal of excavated soil would incur a disposal cost of roughly 6.675 million RMB (based on a local rate of 200 RMB/truck). In contrast, preparing the novel grout from on-site shield muck would consume about 2336.7 m^3^ of muck (for a total grouting volume of ~4428.8 m^3^), thereby avoiding approximately 467,000 RMB in disposal expenses.

In summary, the novel synchronous grouting material delivers substantial direct cost savings while cutting environmental disposal costs through the productive reuse of shield muck, highlighting its clear economic and environmental benefits.

## 5. Conclusions

To overcome the prevalent issues of low early strength, slow setting, and inefficient muck recycling in conventional synchronous grouting, this study designed an advanced grouting material. The formulation utilizes shield muck (containing 71.47% quartz, 15.3% calcite) as the principal component, cement as the binder, and a slag–TMS-Na system as a composite activator. A comprehensive evaluation of the grout’s workability, mechanical performance, and microstructure was carried out, the synergistic mechanism of the additives was elucidated, and the material was further validated in field applications. The principal conclusions are as follows:

1. The optimal mix proportion for the novel synchronous grouting material consists of 60.8% shield muck, 23% water, 8% cement, 8% slag, and 0.2% TMS-Na. This formulation exhibits a compressive strength of 1.52 MPa at 3 days and 4.89 MPa at 28 days, and achieves an impermeability grade of P10. Relative to conventional grout, it offers substantially improved performance: fluidity is increased by 47 mm, setting time is shortened from 11 h to 3.5 h, and early and later-age compressive strengths are enhanced by approximately 97.4% and 93.3%, respectively.
2. The hydration of the novel material over 28 days led to substantial microstructural refinement relative to the conventional grout, characterized by a 58.12% reduction in average pore size (34.55 nm decrease), an 11.69% lower porosity, and a pore structure where intermediate and micropores constitute 66.22% of the total volume. These changes are primarily due to TMS-Na, which accelerates hydration and promotes the generation of additional C-S-H gel, Ca(OH)_2_, and CaCO_3_ crystals. This contributes to a denser and chemically more homogeneous microstructure, ultimately enhancing the overall material performance.
3. MIP, SEM, FTIR, and TG analyses indicate a reaction mechanism consistent with organosilane behavior under alkaline conditions. The hydrolysis of TMS-Na is expected to yield reactive intermediates such as trimethylsilanol. These species can undergo condensation reactions with the -OH groups on dissolved aluminate (${AlO}_{4}^{5-}$) or silicate (${SiO}_{4}^{4-}$) ions derived from the slag, forming stable Si-O-Al or Si-O-Si covalent bonds. This process facilitates the formation of an organic-inorganic hybrid three-dimensional network. Simultaneously, the highly alkaline environment provided by TMS-Na promotes disruption of the slag’s glassy structure, releasing additional ${SiO}_{4}^{4-}$ and ${AlO}_{4}^{5-}$ ions. These ions subsequently participate in pozzolanic reactions with Ca^2+^ to form calcium silicate hydrate (C-S-H) and calcium aluminosilicate hydrate (C-A-S-H) gels. The formation of these gels contributes to pore filling, thereby enhancing the mechanical strength of the grout.
4. The novel grouting material enables a shield muck utilization rate of up to 60.8% and reduces material costs by 71%. A practical application in the remaining 692 rings of Changsha Metro Line 7 is projected to yield total savings of approximately 617,000 RMB, comprising about 150,000 RMB in direct material costs and 467,000 RMB in muck transportation and disposal expenses. It should be noted that geological heterogeneity along the tunnel leads to variations in muck composition (e.g., clay content, particle gradation, moisture content), which can affect the consistency of slurry workability (e.g., fluidity) and mechanical properties (e.g., strength, durability). Therefore, enhanced muck characterization and stricter quality control are required. Future work should focus on developing real-time muck monitoring and adaptive mixture proportion adjustment systems to achieve intelligent production and ensure consistent product quality.

## Figures and Tables

**Table 1:** Chemical composition of grouting raw materials.

Type	SiO_2_/%	Al_2_O_3_/%	CaO/%	Fe_2_O_3_/%	MgO/%	K_2_O/%	Na_2_O/%	TiO_2_/%	P_2_O_5_/%	SO_3_/%	CO_2_/%
Cement	20.22	6.2	63.21	4.86	0.85	1.02	-	0.3	0.1	3.24	-
Shield muck	71.47	7.38	9.6	0.45	0.75	0.48	0.36	1.16	-	-	-
Slag	31.52	14.75	38.45	0.32	7.79	0.31	0.42	1.21	-	-	-
Fly ash	40.43	29.21	10.25	12.72	0.4	2.22	-	2.24	0.84	1.69	-
Bentonite	58.22	17.52	3.12	14.38	0.86	3.77	-	1.69	0.44	-	-
Sand	56.92	11.35	11.05	12.45	1.61	4.12	-	1.84	0.66	-	-

**Table 2:** Orthogonal experimental mix proportion.

Specimens	*A*	*B*	*C*	*D*	Shield Muck
A1	7	7	0.1	23	62.9
A2	7	8	0.2	24	60.8
A3	7	9	0.15	25	58.85
A4	8	7	0.2	25	59.8
A5	8	8	0.15	23	60.85
A6	8	9	0.1	24	58.9
A7	9	7	0.15	24	59.85
A8	9	8	0.1	25	57.9
A9	9	9	0.2	23	58.8

**Table 3:** Cost comparison of conventional and novel synchronous grouting materials.

Item		Mass Proportion (%)	Dosage (kg/m^3^)	Unit Price (RMB/t)	Total Price (RMB/m^3^)
Conventional synchronous grouting materials	Cement	10.6	190	360	68.4
	Sand	36.3	650	150	97.5
	Fly ash	19.6	350	220	77
	Bentonite	5.6	100	600	60
	Water	27.9	500	5	2.5
	Total	100	/	/	305.4
Novel synchronous grouting materials	Cement	8	141	360	50.76
	Slag	8	141	240	33.84
	Shield muck	60.8	1072	/	/
	Water	23	405	5	2.03
	TMS-Na	0.2	3.5	950	3.33
	Total	100	/	/	89.96

## Data Availability

The original contributions presented in the study are included in the article, further inquiries can be directed to the corresponding authors.
